# (Un)timely care: findings from the Waiting Times project

**DOI:** 10.12688/wellcomeopenres.22556.1

**Published:** 2024-08-21

**Authors:** Lisa Baraitser, Kelechi Anucha, Jocelyn Catty, Stephanie Davies, Jordan Osserman, Laura Salisbury, Michael J. Flexer, Martin D. Moore

**Affiliations:** 1 School of Social Sciences, Birkbeck University of London, London, England, UK; 2 School of Humanities and Social Sciences, Leeds Beckett University, Leeds, England, UK; 3 Child and Adolescent Mental Health Service, Oxleas NHS Foundation Trust, Dartford, England, UK; 4 Department of Social, Therapeutic and Community Studies, Goldsmiths University of London, London, England, UK; 5 Department of Psychosocial and Psychoanalytic Studies, University of Essex, Colchester, England, UK; 6 English and Creative Writing, University of Exeter, Exeter, England, UK; 7 Department of Archaeology and History, University of Exeter, Exeter, England, UK

**Keywords:** time, temporality, care, NHS, waiting, crisis

## Abstract

There is a historic crisis in waiting times in the UK’s National Health Service. Crisis brings both a call for judgement – a response to the question ‘what has gone wrong?’ – and a call to action, such as better management, more resources, strategies to mitigate staff burnout, or even a shift in access commitments to reduce demand. However, not all forms of waiting are a sign of service inefficiency or failure, or a form of abandonment or lack of care. Instead, we argue that all healthcare entails waiting, and other forms of elongated time such as pausing to observe, staying alongside patients at end of life, enduring or even encouraging the repeated presentations of those with medically unexplained symptoms, delaying treatment to see what time will bring the situation, or stopping treatment as an ethical intervention. In this paper, we offer three examples of care practices that require waiting and that take place ‘fugitively’, in the ‘seams’ of the NHS, demanding considerable patience on the part of patients and healthcare workers: care for the chronically unwell in general practice; care of young people in mental health crisis; and care for trans and gender-questioning young people. Cutting across the ideological processes of marketisation and provision rationalisation and the linear models of time that have dominated health policy in the past forty years, we argue that understanding ‘timely’ care as relational, interdependent, and paradoxically ‘untimely’ enables a vital recasting of what it means to wait in and for care in the NHS.

## Plain language summary

There is a historic crisis in waiting times in the UK’s National Health Service. Crisis brings both a call for judgement – a response to the question ‘what has gone wrong?’ – and a call to action, such as better management, more resources, strategies to mitigate staff burnout, or even a shift in access commitments to reduce demand. However, not all forms of waiting are a sign of service inefficiency or failure, or a form of abandonment or lack of care. Instead, we argue that all healthcare entails waiting, and other forms of elongated time such as pausing to observe, staying alongside patients at end of life, enduring or even encouraging the repeated presentations of those with medically unexplained symptoms, delaying treatment to see what time will bring the situation, or stopping treatment as an ethical intervention. In this paper, we offer three examples of care practices that require waiting and demand considerable patience on the part of patients and healthcare workers: care for the chronically unwell in general practice; care of young people in mental health crisis; and care for trans and gender-questioning young people. We argue that understanding ‘timely’ care as relational, interdependent, and paradoxically ‘untimely’ enables a vital recasting of what it means to wait in and for care in the NHS.

## Introduction

In July 2016, the father of one of the authors was admitted to hospital. She rushed to be with him, but by the time she got there he was in state of acute delirium. Every ten minutes or so he would grab her hand and say, ‘Come on. Let’s go’; and then, ‘What are we waiting for?’ She explained that he was very ill and that the doctors needed to find out what was wrong. But as for what, exactly, they were waiting, there were no easy answers: for the medical situation to become clearer; for things to unfold; for time to pass? She found herself repeating: ‘You need to be patient’. ‘Patient?’ he would reply. Her father could see well enough that he was being treated as some sort of patient, but he was struggling to understand how the waiting being asked of him represented a form of care that felt ‘timely’.

This experience had a particular significance to us as a group of health humanities researchers working to investigate the role and meaning of waiting in healthcare, and particularly in the UK’s National Health Service (NHS). The author involved accompanied her father through his hospitalisation in the NHS, applications for social care, and then the final phase of his life, getting to see up close how waiting can be a screen for an over-stretched service – its inefficiencies, insufficiencies, and at times even its failures to care. But she also glimpsed a rather harder to conceptualise aspect of waiting: the elongated yet undramatic forms of care given when nothing very much appears to be happening; when a situation develops, or fails to develop; and in the face of a future that is radically uncertain and when waiting is all that remains. She came to see how care was indeed being offered by health professionals and by the service itself, and that care was in part being collaboratively ‘made’ through these stretched out periods of time. Because of the pervasive difficulties with noticing and conceptualising waiting *as* care, however, we came to understand that we urgently need new vocabularies and concepts that might help patients, clinicians, families, and carers to situate and communicate the meaning of their waiting. It is this that has informed the development of a collaborative research project, *Waiting Times*.

Running from 2018 to 2023, the time of the project was fractured by two major social and political events: the global coronavirus disease (COVID-19) pandemic, and, in the UK, an escalating crisis in NHS waiting times. On the one hand, the pandemic pushed waiting, care, vulnerability, racialised violence, inequality, interdependence, and mourning forcefully into public consciousness, causing us all to think again about waiting, care, and forms of neglect ( Baraitser & Salisbury, 2020). On the other, after the acute phase of the pandemic, the public discussion about waiting and healthcare became more and more focused on just one issue: NHS waiting times. By October 2022, as the *Waiting Times* project drew to a close, 7 million people were waiting for elective care, and an early report by the National Audit Office (2021) described a possible 12 million-strong waiting-list by March 2025. By September 2023, the figure from NHS England was 7.68 million. The convergence of the pandemic with the real terms cuts to government spending on healthcare, an increasingly ageing population with complex needs, a lack of political will to reform the social care system, and an increase of chronic physical and mental health conditions for those most affected by more than a decade of austerity, meant we found ourselves in what was being characterised as the biggest ‘crisis in waiting’ in the health service’s history ( Salisbury *et al.*, 2023).

Care has a distinctive relation to timeliness. To wait in pain or when treatment is urgently needed is not only difficult but can be dangerous. Once in labour, for instance, a pregnant person cannot wait for the kinds of care needed for the safe birthing of a baby; the hallmark of good stroke care is that time is of the essence; uncontrolled post-traumatic bleeding requires prompt measures to avoid preventable death. ‘Timely’ care, in these contexts, means calibrating waiting very precisely in relation to urgent need. But what does timely care look like outside of acute situations and emergency medicine? We have argued elsewhere that effective care recognises both the demand for active and often urgent interventions and the simultaneous importance of forms of waiting, delaying, slowing, pausing, repeating, stopping, or simply allowing time to pass – what we are calling here ‘untimeliness’ ( Salisbury *et al.*, 2023). Of course, healthcare practitioners know well that waiting and care are not opposed to one another and that where care exists, so does waiting. It is there ‘in the extended time needed for therapy or therapeutics to work; in the watchful waiting before or after diagnosis; or in the time that stretches through remission, relapse, or palliative care’ ( Salisbury *et al.*, 2023, n.p). Here, waiting *with* or simply continuing to be available becomes the key way of supporting health ( Baraitser & Brook, 2020; Salisbury & Baraitser, 2020). Timely care, in these contexts, entails reckoning with the complexity and essential untimeliness of attending to the shifting and uncertain needs of an other in the face of an unknown future and with resources that are always, in some way, limited.

How time is ‘spent’ in healthcare, by both patients and practitioners, is conditioned by wider historical, social, and political forces that govern and structure time ( Burges & Elias, 2016) and determine whose time is important and whose can be wasted ( Sharma, 2014); how time is experienced ( Baraitser, 2017); and what ‘timely’ comes to mean. If we look to the history of the NHS, for instance, we see that versions of untimeliness were baked into the system from the start: the existence of private pay-beds for wealthier patients being a case in point ( Bar-Haim *at al.*, 2023). But although waiting has always played out unevenly, it was also collectivised in the new health service, becoming a meaningful part of a shared social project. For a period, at least, waiting together for healthcare that had previously been inaccessible to many had a social meaning – an idea and promise that has continued to remain alive in the cultural life of the UK ( Thomson, 2022).

Given the collective waiting embedded in the history of the NHS, there may be a particular ‘NHS-ness’ ( Bar Haim *et al.*, 2023; Salisbury *et al.*, 2023) about the relationship between waiting and care that is not captured by the waiting times crisis headlines. This ‘NHS-ness’ was invoked positively during the first lockdown of the COVID-19 pandemic, when people were asked to wait as an act of collective social care in the name of protecting a healthcare service in which they were invested, both materially and morally. Of course, the patience of ordinary people quickly wore thin when it became clear that the collective willingness to wait for the NHS was not shared by many of the politicians who had imposed lockdown restrictions. ^1^ Furthermore, although everyone has to wait, at some level, in and for care, this is not an equitable experience and frequently reproduces broader social injustices ( NHS Race & Health Observatory, 2022). The NHS-ness of waiting often plays out, we suggest, in having to endure time that doesn’t feel like it is passing or flowing well. Nevertheless, in these experiences of waiting, care emerges in and through ‘stuck’ time, in what we term the ‘seams’ of the NHS: in processes, practices, exchanges, transactions, and relational moments that run alongside more obvious care pathways. Frequently characterised by ‘negative’ valences – by exhaustion, anger, and even despair – care takes place in the arcs rather than nodes of a pathway, even when change itself may be difficult to spot. By staying alongside or enduring time with patients ( Baraitser, 2017), care may be perceived, even when nothing seems to be getting better.

The *Waiting Times* project has undertaken a multi-strand investigation of the relationship between waiting and healthcare, drawing on health humanities and social science frameworks that situate both temporal experience and healthcare within an historical context, while drawing on literary, psychosocial, and engaged research methodologies to understand contemporary experiences of waiting within healthcare. A key finding is the difficulty of tracking waiting as a meaningful object, yet the importance of not losing sight of the ways that pausing, delaying, slowing, and stretching (or making the most of) time, for both practitioners and patients, is as essential to timely care as crisis interventions. Indeed, we have suggested that there are significant risks in focusing almost exclusively on the reduction of waiting times and waiting lists as a way to support the health of the NHS and its capacity to care for its patients and workforce ( Salisbury *et al.*, 2023).

In this paper, we elaborate these ideas by presenting three vignettes taken from ethnographic and practitioner-led research carried out in the UK’s NHS: in general practice, in child and adolescent mental health services, and in a gender identity service for young people. These sites are particularly resonant as they allow us to see how care is made relationally in situations where patients and practitioners may feel that nothing is moving on in a timely way. We argue that these sites show practitioners ‘caring on’ in order to offer something that is ‘timely’, in the more expanded sense we have begun outlining here, by making time in situations where it appears to have ground to a halt. Drawing attention to the time of ‘caring on’ should not be mistaken for a suggestion that the NHS is currently adequately funded; but it is to suggest that the complex conditions under which timely care is made, and the resources required to support these care practices, demand conceptualisations of time that are not simply linear and finite. Instead of seeking to address the ‘crisis’ of the NHS by moving people more quickly through the system, reallocating time to address ‘shortfalls’ to reduce waits to access care, or even abandoning the social commitment to a universal service, we would do well to pay attention, first, to how time and care continue to be made in the ‘seams’ of the current system. It is only by reckoning with this essential ‘untimeliness’ that we might then be able to conceptualise interventions in the NHS that are themselves timely and that continue to underpin its social mission.

To conceptualise this way of understanding the relation between care and waiting, we use the notion of ‘fugitive care’ developed by one of the co-authors, Kelechi Anucha (2023). This concept considers how care is distributed unevenly and the racialised and minoritised work of inventively carving care out within stuck time. Drawing on this, we argue that we can glimpse care in the ‘fugitive’ practices of practitioners, patients, and services. These practices are not simply time-bound, with care being constrained by time's pressures; instead, they use time in relational ways to enable more care to be made.

## Fugitive care

Anucha defines ‘fugitive care’ as the ways in which care can be ‘inventive, improvised and endlessly challenging, taking place in excess of sanctioned clinical and social pathways’ ( Anucha, 2023: 12). Fugitive care names the strategies, kinship claims and allegiances of embodied care that emerge within marginalised communities for resisting and negotiating structures of, and pathways through, institutions ( Anucha, 2023: 12), whether political or medical. Anucha articulates how these practices of care enable mainstream health care services not just to carry on, but to ‘care on’ in situations of intense time and resource pressure.

In her work, Anucha brings care into contact with a concept of fugitivity originating within Black studies ( Harney & Moten, 2013; Hartman, 2019; Hartman, 2021; Sharpe, 2016). Fugitivity names a way of being in the world, embodied first by ‘the shipped’: those racialised as black and subjugated by the Transatlantic slave trade ( Moten, 2018). Fugitivity, however, may also be conceived as a form of ‘escapology’ ( Emejulu, 2022) that opens a space of possibility in which dreaming, fantasy, speculation, hope, and collectivity gain heightened value and potency ( Halberstam, 2013). ^2^ Dreaming constitutes a raw form of care, Anucha argues, but it is often the only care possible for people to offer to one another in unliveable conditions. Such dreaming runs alongside forms of fugitive care in action, as material aid is improvised in the absence and dangers of ‘proper’ medical attention, offering a witnessing presence and emotional succour that makes it possible for life-in-death to go on.

These spaces of fugitive care may materialise in unexpected and unorthodox circumstances. Christina Sharpe (2016) describes her brother Stephen’s last moments, relating how friends and family gathered around his bed, ‘shared stories, played music, laughed and told [him] how much [they] loved him’ ( Sharpe, 2016: 10). Even in this space of care, however, Sharpe registers an awareness of the how the US healthcare system and medical racism make it necessary for the family to work hard to ensure Stephen received the palliative medication he needed.

Anucha argues that it is worth asking how we can productively bring fugitivity – a concept originating in the context of an overwhelmingly US-oriented Black studies field – into dialogue with discussions about how caring takes place within the increasingly politicised terrain of the UK’s NHS. In the three vignettes that follow, we use the idea of ‘fugitive care’ as a framing device, turning a spotlight on the often improvised and inventive care that takes place in an overstretched NHS. Through these vignettes we attempt to track the elusive object of waiting as it is offered, and at times experienced, as a mode of careful attention. In the face of what is easily represented as a failure of care, we note how giving time to a situation can turn out to be a collaborative endeavour that produces rather than reduces care.

## Elongated waiting in general practice

Stephanie Davies’s study of waiting, staying, and enduring in contemporary General Practice investigates forms of care that are precariously maintained in situations in which nothing appears to improve ( 2023). These might include ongoing care – or what we are calling caring on – for what is in danger of being neglected during periods of crisis. This includes care for chronic states in an outcome-orientated healthcare setting; for inevitable physical and mental decline and degeneration; and for the NHS itself after a loss of faith in its promise of a better future. In these situations, activity that appears the least productive in terms of outcomes can sometimes be the most responsive. Given the rise in long-term health conditions that disrupt clinical ideas around the acute and the chronic, the communicable and non-communicable, combined with a steady withdrawal of state funding for long-term support, there is a case for arguing that these are the modes of care most needed in the present, rather than crisis interventions aimed at restructuring the system to reduce waiting.

It is well accepted that many of the conditions cared for in contemporary NHS general practice now are chronic ( Williams & Law, 2018), ‘treatment resistant’ ( Thomas *et al.*, 2013), complex ( Cassell *et al.*, 2018), or self-limiting (resolving in their own time) ( Fielding *et al.*, 2015). This means that it is not unusual for whole episodes of clinical care to revolve around situations that patients and practitioners are unable to change or move on from. This kind of healthcare falls outside of the scope of preventative medicine where the emphasis is on striving to safeguard the future of a population perceived as ‘constantly at risk’ ( Armstrong, 1985: 664). It also falls outside the linear flow of medicine conceived in treatment pathways, with their constant movement towards a goal.

While this shift in general practice’s core work suggests the need for longer-term support and more open-ended ways of responding to need, the infrastructure for providing continuity of care in the NHS has been eroded in recent decades. Clare Gerada, President of the Royal College of General Practitioners (GPs), writes about how, during her last day working as a GP on call, she was called out to see several patients in her extended London catchment area, none of whom she had ever met before ( Gerada, 2022). What these patients had in common, besides old age, was that they were suffering from serious illnesses that could not be reversed or improved with simple interventions such as prescribing painkillers or antibiotics. After every visit, Gerada left feeling saddened by the experience of having so little to offer them.

Each patient I saw that day was a stranger, and each contact an isolated encounter. We would never meet again. They were single episodes and, unless I made an error resulting in a complaint (and this is a constant anxiety), I would never know the outcome of my actions.

In Davies’s study, when she asks Ben, a GP, what he believes his patients are expecting from him in situations where they have reached the limits of what medicine is able to do, he tells her about the case of Joe, a ‘white working-class man’ who is dying of cancer:

Ben does not know Joe, he has not come frequently to the surgery. After a visit to see Joe at home, Ben describes feeling as though he has ‘fallen into a scene’ and is having to make sense of what he is there to do. In recent months, Joe’s body has grown weaker from metastatic cancer. The signs are visible. Ben says that the experience of being a doctor who is ‘thrown into so grave a context’ as the home of a dying man sets up an encounter in which he feels as though he is playing out a centuries-old role. In his words, ‘you are the representative of a profession for whom this is very well-trodden ground’. So, when he sees Joe lying in his bed, he tries to take hold of what has to be done. He thinks, ‘we’re in a new phase: the dying phase’. They discuss palliative care drugs and Ben describes feeling close to Joe during these moments and wanting to be more involved between now and the time of death: ‘I feel a kinship with him... a great sense of privilege to be his doctor… to be trying to hold on to his sense of autonomy and ownership over decisions.’ He hopes that Joe will still be at home when he comes back for follow-up in ten days’ time, although he struggles to say what exactly he believes could be achieved by seeing him again. He says that it might have something to do with bearing witness: ‘I can bear witness... be around for him if he wants to talk.’ ( Davies, 2023: 182)

John Berger places this elusive gesture of staying close when the time of death is drawing near in the medical tradition of ‘witnessing’: ‘if [the doctor] cannot cure us, we are also asking him to witness our dying’ ( Berger, 1967: 62). Ben does not necessarily share Berger’s strong conviction about the benefits of bearing witness, but it is something that he can do, if Joe wants him to. This is all happening outside the context of anything like a long patient-doctor relationship or a standard clinical pathway. It is a situation which Ben feels he has been thrown into and is having to make something good out of. He reflects that it is perhaps not what he knows, or what he can do that can be of use to Joe and those around him now. He has already reached the limits of what more pharmaceutical intervention can offer. It is more just the fact of his being there, inhabiting the role of somebody who is making an offer of time, in the hope that this will be picked up and transmuted into something meaningful. In effect, he is waiting to see if he can be of any use to Joe. Whether he can be or not, depends on the potential for Joe to take up his offer to, in Ben’s words, ‘use me’.

‘Use’ is a problematic term for medicine, although it has its own small tradition in general practice, after being introduced into the NHS by psychoanalysts Michael Balint and Donald Winnicott in the 1950s ( Balint, 1957; Winnicott, 1969). For Ben, the hope that he may be of some use to Joe and his family seems risky, as he extends to Joe something that may or may not be the thing that is needed or wanted. The offer to ‘use me’ in this context can never be sure of its effect. It has the potential to fail, to be rejected, to complicate things or to prolong a bad experience of healthcare. It might not even feel like much of a choice. Whereas a vascular surgeon or a respiratory consultant can apply their medical know-how to Joe’s electronic record and decide not to get involved, Ben does not have the same option to decline to respond to what he cannot act on. Gerada recalls that in the past, there had seemed to be more places for people to go when they needed time to be ill for a while, or to wait whilst dying: ‘back then, I had access to a cottage hospital where I could admit patients just because they were “off their feet”’ ( Gerada, 2022). In the absence of the structures needed to support the sharing of time that has stopped flowing, ‘use’ for people like Ben and perhaps for Joe too, also implies wear, as in the wearing on (or wearing out of) a person or a workforce that happens when they are used or employed for purposes not necessarily their own, or not what they intended.

During times of crisis, offering time to a situation that will probably not change could easily appear wasteful. But what if the very capacity to be moved to care about someone or something depends on time being allowed to pass, without necessarily knowing in advance what good, if any, will come of it? The philosopher of science Vinciane Despret writes: ‘those who wait and those who wait on (or attend) sometimes become attuned and learn to be affected — that is, to care’ ( Despret, 2004: 131). In Davies’s ethnographic work, what comes to the surface is the potential of this ‘learning to be affected’, which can make care in cases like Joe’s, if Joe is in a position to ‘use’ Ben’s offer of waiting. Together this undermines the assumptions implicit in care plans which aim only at management, progression, and ‘satisfaction’. In NHS general practice today, it often happens that neither patient nor practitioner knows how long the experience of waiting for something to end, or to give way, is likely to go on for, or whether the help that is offered in the meantime is going to make things better or worse. The offer of time by itself might never feel like it is enough, yet we understand it as an attempt, under pressure, not to move on too quickly, but to stay long enough to become attuned to what the other might still need.

## Waiting in adolescent mental health care

Jocelyn Catty is a Child and Adolescent Psychoanalytic Psychotherapist in a busy NHS Child and Adolescent Mental Health Service (CAMHS). In her clinical work with adolescents, she is acutely aware of the urgencies of time and care, that she links, in part, to the pressures adolescents face. Adolescence is understood in contemporary Western cultures as a transition in time, between childhood and adulthood. Half in, half out of childhood, young people are asked to hold both their own and adult hopes and anxieties about the future. ‘Janus-faced’ ( Waddell, 2018), they occupy a position that points them both forwards and backwards. If adolescence may be said to be a crisis *in-and-of-time* ( Catty, 2021b), these pressures converge when adolescents go into mental health crisis. Inter-generational trauma, often mediated by parental mental ill-health, domestic violence, neglect, and attachment difficulties, is known to play a key role in the aetiology of adolescent depression ( Cregeen *et al.*, 2016). Yet dread of the future, *or pre-traumatic stress* ( Kaplan, 2020; Saint-Amour, 2015; Salisbury, 2020) ^3^ figures just as powerfully for young people at this pivotal stage. On the brink of a future that they may see as no future at all, traumatic pasts, whether their own or inter-generational, invest their looking-forwards with grief and dread: that the trauma may be repeated ( Freud, 1914) or that the future may already be cancelled. Thus, the past may condition them to read the future through the lens of their own or their ancestors’ traumatic histories, leaving no sense that the future is worth living. For those in extreme crisis, the suicidal act, the overdose, or other act of deliberate self-harm, may be seen as calling a halt to time, often in a way that is terrifying for the young person and those tasked with caring for them.

That the current generation of young people may be under extraordinary pressures is reflected in soaring referrals to CAMHS both before the COVID-19 pandemic ( Nuffield Trust, 2019; Therrien, 2018) and since ( Gregory, 2023). The lockdowns of the pandemic, in which adolescent pressures gave the lie to the idea that time could be suspended ( Catty, 2021a), are known to have led to a marked deterioration in adolescent mental health and increase in psychological distress, particularly for those young people already struggling ( Branje & Morris, 2021; Kiss *et al.*, 2022; Li *et al.*, 2022; Luijten *et al.*, 2021). The increase in adolescent mental health presentations has also been linked to the impact of social media ( Karim *et al.*, 2020) and exam stress ( Steare *et al.*, 2023), to which may be added the impact of ongoing structural racism ( Simon, 2023) and discrimination, including that experienced by LGBTQ+ young people ( Mustanski *et al.*, 2016). Anxiety about climate change ( *Lancet*, 2022) also features: one alternative narrative of the future figures inter-generational trauma as the result of ecological violence wreaked by one generation upon the next, prompting a ‘cry for help’ ( Baraitser, 2020; Thunberg, 2019). While psychiatric classifications of depression reliant on the notion of ‘irritability’ have been shown to fail to do justice to young people’s rage ( Midgley *et al.*, 2015), models of depression that invoke ideas of stuck, suspended or impeded time ( Minkowski, 1970; Salisbury & Baraitser, 2020) may similarly overlook the *Sturm und Drang* of the adolescent crisis ( Catty, 2021b).

Yet if adolescent mental health crisis appears to threaten catastrophe, it also brings the potential for development, growth, and psychic change. For when a young person calls a halt to what they perceive to be a relentless march towards a bleak future, then, provided that the suicidal action remains uncompleted (the threat of this tragic outcome casts a significant shadow over mental health practice, Catty, 2021b), they create a crisis-point in the sense of a point of pivot or transition ( Roitman, 2016). This opens a time in which matters may rest before moving forwards or back. They thus ask for attention and intervention in a way that has the potential to open up time for care.

To illustrate this, Catty describes a clinical situation with a young person she calls ‘Layla’:

Layla was referred to her local CAMHS at the age of 12 because of her low mood and anxiety, which had got worse when she started secondary school. By the time she was seen at age 13, she had been waiting for over a year, and had started self-harming by cutting herself regularly. Clinicians elicited from her concerned mother and stepfather a history of domestic violence perpetrated by her birth father in her early years, and a sense that she was an unofficial ‘young carer’ for her depressed mother. Layla herself engaged in a rather perfunctory way with a series of appointments with clinicians, who felt they could not elicit any sense of optimism from her about the future, nor any meaningful account of why she was self-harming. The appointments had no apparent effect on her mood or self-harm. She was then referred to the team’s psychotherapist. The day before she was due to meet the psychotherapist, however, Layla took a significant overdose of painkilling medication; frightened, she told her mother and was admitted to hospital.
  When Layla was discharged from hospital and the outpatient sessions with her psychotherapist commenced, the therapist was able, gradually, to talk with Layla about the timing of this overdose, picking up on a sense of ‘too-lateness’: that a space for care and attention was being provided, but too late to be of use. Layla for the first time brightened. She admitted, ‘I think this therapy is too late for me’. From this moment of contact between patient and therapist, a genuine therapeutic relationship was able to grow. Layla started to talk more freely, not just about feeling low, but about things that annoyed her - at school, or even at home – and her worries about the future. Gradually, she dared to admit her mixed feelings about being her mother’s ‘carer’. Gradually, her self-harming stopped; much later, she was able to tell her therapist that she felt proud of having ceased to resort to this way of signalling her distress.

There is a crisis in the system of care for young people like Layla. When a 12-year-old girl is left waiting over a year for help, there is an urgency that merits newspaper headlines about waiting times. In this timeframe, the chronic crisis of depression and anxiety acquires the greater urgency of self-harm. But responding ‘urgently’ and in a straightforwardly timely way, while important, is not the only solution here: necessary, but not sufficient. For successive appointments fail to reach Layla, who continues to believe that care by professionals, perhaps by anyone, is ‘too late for me’: a devastating belief that she acts on in a dangerous overdose. This belief cannot be shaken until it is recognised, with time, by the psychotherapist, and named. Then, Layla can experience her most difficult and frightening feelings being recognised and tolerated, in an experience Bion (1962) conceptualised as *containment*. Once she accesses this containing space provided by her psychotherapist through the recognition of her fears, the offer of time that *is* psychotherapy ( Salisbury & Baraitser, 2020) becomes meaningful. Once again, we could say Layla can ‘use’ the offer of waiting, and this in turn produces a collaborative form of care, which mitigates her conviction of ‘too-lateness’ and opens up a sense of the future.

## Untimely care: the case of GIDS

In our third vignette, we describe ethnographic work undertaken by Jordan Osserman at the Gender Identity Development Service (GIDS) within the Tavistock and Portman NHS Foundation Trust. At the time the research took place, GIDS was a service with a ballooning waiting list. Waiting for gender care as a young person is extremely difficult, according to both service users and practitioners working with them ( McKay *et al.*, 2022), suggesting that the kinds of *waiting with* that are possible are themselves time-sensitive and often experienced as untimely. These untimely interventions may feel too late for the young person concerned if they experience being held up or delayed by a service that takes years to see them, and then appears to offer more waiting in the form of ‘thinking time’ ( Barnes, 2023), when what the young person feels they need is treatment. Meanwhile clinicians may worry about interventions appearing too soon on a scene where understanding cannot yet be fully grasped – with gender development creating its own untimeliness and disruptions in development and sequence.

Osserman’s study with GIDS, however, turned out to track not just how the service managed experiences of waiting, but how waiting was punctuated by the immediacy of crisis. The study began with one crisis and ended with another: the onset of the COVID-19 lockdown in 2020 and then the announcement of the imminent closure of the service in July 2022. In between, the service faced numerous additional crises including: the UK High Court ruling on *Bell v Tavistock* (2020); the subsequent overturning of this ruling ( *Bell v Tavistock 2021*); a rating of ‘inadequate’ from the Care Quality Commission (2021); the publication of an interim report on gender services for young people, produced by the Cass Review, which was used by their NHS commissioners to underpin the decision to close the service (2021), and in March 2024 the actual closure of GIDS and the publication of the final report from the Cass Review (2024). The closure of GIDS and replacement by new services was elongated, following NHS England’s failure to meet their Spring 2023 deadline, whilst the GIDS waiting list was itself transferred to a third-party provider – leaving the existing service in a state of uncertainty, waiting and staff attrition at the time of the ethnographic work. GIDS therefore seemed simultaneously a site of acute and chronic crisis, many instances of which were inseparable from questions of time. The question of whether GIDS makes patients wait too long, or not long enough, for instance, has been at the heart of public debate ( Osserman & Wallerstein, 2022). Alongside the Cass Review (2024), a journalistic investigation of the service, *Time to Think,* cast serious doubts on whether GIDS really provided the thinking time it claimed to champion ( Barnes, 2023). However, these investigations of GIDS have themselves faced accusations of anti-trans bias ( Hayes, 2023; McConnell 2024). ^4^ Osserman noticed that the service’s complex attempts to ‘care on’ amid overwhelming crises had parallels with the temporal complexities faced by its service users.

We offer here a composite and anonymised vignette from an ethnographic observation of a supervision meeting, as a further elaboration of this idea. At GIDS, supervision meetings are spaces where clinicians can think about the young people in their care, discuss treatment plans with one another, and talk about the ways the pressures of the clinical work may be affecting them and their work with young people:

In the meeting, clinicians described working with a young person and her family who were insistent that, if puberty blockers were not prescribed soon, the young person would commit suicide – tragically, this can be a feature of this work. The young person described experiencing gender dysphoria from early childhood and experiencing puberty as ‘wrong’. The clinicians said they struggled to maintain GIDS’s protocol, which required several more assessments before a referral could be made to the endocrinology service, although they did maintain it. In the penultimate assessment session, they discussed with the child and family the practicalities of puberty suppression – the pros and cons, the side effects, and so on. The next session, when a referral would have been made, the young person announced she was ‘de-transitioning’. As the clinician described it, ‘ *She felt she had understood her gender dysphoria within the context of misogyny. She wanted to escape from the pressure of being female. She said she avoids reading too much about detransition, because she supports trans rights; and she values the time she has lived as male. She told us about how she likes to be loud and opinionated, to share her thoughts, and this seemed more acceptable to do when living as a boy. She said she now has the confidence to be a loud woman.’* The patient did not turn up for subsequent appointments.

The clinicians share how difficult this case was for them, and their wish to have a reflective space to consider this work which, it appears, has ended. The conversation centres around the appropriateness of ‘holding the line’ on GIDS’s assessment protocol – a war metaphor which also evokes the embattled condition of the service. One asks whether the sharing of practical information about puberty blockers – making medical treatment appear imminent – ‘took the fight out of it’, allowing the young person to reflect on herself more deeply. There appears significant relief that the assessment seemingly enabled the young person to decide she did not want to pursue a medical pathway prior to the referral being made. However, one clinician wonders: ‘what if we *had* prescribed puberty blockers, and then the young person changed their mind? Isn’t it cis-normative of us to assume that the worst possible thing is to begin and then stop transition?’

There is untimeliness permeating this scene at multiple levels. On one, there is the child, whose experience of gender dysphoria is unbearable, but who seems to reach a retroactive insight about her gender identity at the very point when medical treatment becomes within reach: *I am an opinionated woman*; or rather, *I have needed to live as a male in order to become the opinionated woman that I always have been*. It appears the language of ‘de-transition’ fails to capture something more temporally complex here: not a ‘return’ to an original gender so much as the making of a new one, through a revisiting of the past occasioned by the anticipation of a possible future. ^5^

Next, there are the clinicians, who were wracked with worry about this young person’s wellbeing and the appropriateness of their protocol whilst working with her. They were certain that once they finished jumping through the hoops, they would be making an endocrinology referral – only to discover, to their bewilderment, that something in this young person had changed over the assessment process without them noticing. We can also note their fear that they might have ‘failed’ to understand the ‘truth’ of this child’s gender dysphoria – no doubt intensified by the tremendous scrutiny that the service is under – and the absence of any sense that they were accompanying a child through a process whose outcome could not be predicted in advance.

Finally, we have the fact of the case presentation itself, taking place in the wake of the young person’s disappearance from GIDS, with staff trying to make sense of what was done here, and what might have been. What kind of clinical supervision involves a patient who is no longer seeking care? We cannot know what ultimately happened to this young person, or why she did not attend subsequent appointments. Will she come to yet another understanding of her gender identity in time? Will she seek gender care again? Seen in this light, the vignette is more an example of the service attempting to take some care of itself in the face of uncertainty.

Another case might illustrate this further. In group supervision, clinicians discuss a 16-year-old trans boy who experienced significant improvement in his mental health following a year on puberty blockers and is now eager to begin hormone therapy. Nervous about approving this next step, the clinicians working with him queried his sense of urgency. Did it prevent him from reflecting sufficiently upon the implications of further medical treatment? The young person explained that while he felt the time was right, he could cope with waiting longer if necessary. Several staff felt reassured by this response. As an observer Osserman detected in this instance a report of a kind of back-and-forth playing out between clinician and young person over the meaning of waiting and urgency. A willingness to wait appears here paradoxically as an indication that waiting can end. It again raises the thorny question of who is being cared for – the child, the service, or the waiting list?

It is clear that many young people have been helped by receiving a referral from GIDS for medical treatment, and moreover that the waiting list for gender care is too long. Rather than comment on GIDS’s protocol or the overall management of the service, here we simply wish to draw attention to the necessity – and difficulty – of attending to the untimeliness involved in care. The uneven, ‘stop start’ temporalities of gender exploration can make care seem, in some sense, impossible. The untimeliness that GIDS is called to account for is perhaps not solely a result of NHS waiting times or service mismanagement (although these are crucial); it may also be a manifestation of something untimely at the heart of gender development itself, which activates cultural anxieties about the movement towards resolution that are then projected onto the figure of the trans or gender questioning child.

Between a young person and a service in both acute and chronic crisis, fugitive care, in this situation, may entail all parties bearing that what is on offer is going, at some level, to miss its mark. Where Layla, in the earlier vignette, can only move forwards when someone understands and validates her cry, ‘it’s too late for me’, gender services for young people are in the difficult situation of having to understand an often-paradoxical communication: ‘it’s too late and too early for me’.

## Caring on: timely and untimely care

Fugitive care in action, as we have been articulating it here, emerges in services under extreme time pressure. Nevertheless, through a significant sensitivity to time and timing, and through an ongoing attention to relationality, care is made in what we have called the ‘seams’ of the NHS. Timely care, in these contexts, emerges paradoxically through attending to the untimeliness that occurs when people allow themselves to become ‘attuned’ to the complex, uncertain needs of others, while being prepared to stay with the rhythms of repeating, returning, delaying, and enduring ( Baraitser, 2017). The possibilities for fugitive care seen in our vignettes are made collaboratively in the untimely time that becomes present between Ben and Joe, if Joe can ‘use’ the waiting that Ben offers him; between Layla and her psychotherapist if the psychotherapist can hear and bear that her care has come ‘too late’; between and among the GIDS staff and their patients, if they can understand that the care on offer from a service already marked for closure is fundamentally untimely, both too soon and too late. If we accept that the NHS is not only a space through which patients move, but a space where people wait and endure together, then what is revealed is a kind of care that never makes it into statistics about waiting times and waiting lists. This is a form of care that holds on to the possibility of reanimating relationality, and that in turn makes more care and more time.

Writing of care in general, although she could be writing about care in and for the NHS, María Puig de la Bellacasa argues that:

even when one cares for the dying, with hope and anxious anticipation, even when care is compelled by urgency to enjoy the fleeting present, charged by past regrets and joys and the weight of accumulated experience, a certain suspension of feelings of emergency, fear, and future projections – and weighty pasts – is required to focus on caring attention. ( 2017: 207)

For Puig de la Bellacasa, holding crisis temporalities in abeyance, even if only temporarily, creates the conditions for care to emerge – a ‘care time [that] suspends the future and distends the present, thickening it with myriad multilateral demands’ ( 2017: 207). If this is so, then within an NHS context, the suspension of overarching narratives of the future – whether utopian or dystopian – might also be paradoxically enabling. This waiting might allow those who care, both in the NHS and for it, to use the ‘depressing time’ ( Baraitser & Brook, 2020) of its chronic crisis not simply to make the most of the little time that is left, but instead to use the care that remains in relationality to expand time: to ‘care on’ fugitively as a way of making the time needed to care.

Anucha alerts us to the need to hold in tension both fugitive care in action, which we have tried to illustrate here, and fugitivity as an imaginary space. This leads us to ask: how can we reframe the waiting that goes on in, and indeed for, the NHS in a way that understands it as a shared, collective practice, rather than something patients do, while doctors act and managers rationalise? This is a particularly timely question when NHS workers have been taking strike action during 2023 and 2024 to demand pay rises to ameliorate the chronic understaffing that threatens the basic conditions required for caring contact to be made in situations of need. If time is represented as a finite resource that must be used efficiently, then ‘streamlining’, speeding up systems, and making cuts to practices that are deemed wasteful seem like obvious solutions. The ideological processes of marketisation and provision rationalisation that have dominated health policy in the past forty years ( Bar Haim *et al.*, 2023) indeed rely on conceptualisations of time as linear and scarce, where moving people more efficiently through the system, reallocating time, or even fundamentally reducing demand, is seen to be what is needed. But if we think *with* waiting, instead of seeking to eradicate it, other possibilities come into view. As two of our co-authors, Stephanie Davies and Martin Moore (2023), have recently argued, throughout its history many of those invested in the NHS – patients, staff, and others – have waited collectively in the name of what the service could or should be. But Davies and Moore draw our attention to a history of activism and demands for change in the NHS as a way of cutting across fantasies of a return to a fully resourced and funded service that in fact has never been a reality. Withdrawing from fantasies about the past and future, they suggest instead that a significant part of preserving what matters about the NHS is being prepared to ‘care on’ without any clear resolution being visible and in the face of significant losses, just as one might in those everyday practices of healthcare where nothing much appears to be happening, as we have drawn attention to here. If we can reckon with the essential untimeliness of care that runs alongside even the most timely interventions, and if we are able to learn from how care goes on being made fugitively when resources are woefully insufficient, it may be possible for all those invested in the NHS’s future to see more clearly, and indeed more imaginatively, the resources required for them to flourish.

## Data Availability

All data underlying the results are available as part of the article and no additional source data are required.
