# Supplementary material for: (Un)timely care: findings from the Waiting Times project
Source: Wellcome Open Res. 2025 Jul 25;9:490. Originally published 2024 Aug 21. [Version 2] doi: 10.12688/wellcomeopenres.22556.2 (PMC12405842; doi:10.12688/wellcomeopenres.22556.2)
Supplement: Peer review history

# Reviewer report: Reviewer response for version 2

**DOI:** 10.21956/wellcomeopenres.27146.r128102

**Published:** 2025-09-02

**Authors:** Lauren Herlitz

**Affiliations:** 1 UCL, London, UK

Thanks for asking me to read this commentary. It is well-written though perhaps could be more concise in the sections relating to the vignettes. The main issue relates to use of the term 'fugitive care' which does not clearly fit with the vignettes or discussion on timeliness. Otherwise, the remaining comments are minor.

*Abstract*

 The abstract is well-written.

*Introduction*

 “… and that care was in part being collaboratively ‘made’ through these stretched out periods of time.” I’m not sure what is meant by this. Who was collaborating to make care?

 Perhaps “two major, inter-related social and political events..”

 The existence of private pay-beds for wealthier patients is not a good example of untimeliness being baked into the NHS, rather it is a good example of inequalities in timeliness. Can you give a different example related to changes in our understanding of timeliness over time?

 Perhaps rephrase “Frequently characterised by ‘negative’ valences – by exhaustion, anger and even despair – care can take place in the arcs rather than nodes of a pathway…” Otherwise is sounds as if care always takes place in the ‘seams’ that you have mentioned when for many patients it doesn’t.

*Fugitive care*

 The concept of ‘fugitive care’ that is defined as “the racialised and minoritised work of inventively carving care out within stuck time”. However, none of the three vignettes show this racialised work (and only the GIDS vignettes focuses on a minoritised group)… they only focus on carving care out within stuck time. This is confusing for the reader. It’s made worse because the explanation of care hints at ‘fugitive’ in the sense of escaping from captivity or being in hiding which doesn’t seem to fit with the vignettes. It’s very unclear how the description of fugitivity fits with the subject of timeliness.

*Elongated waiting in general practice*

 Why is Joe being a ‘white working-class man’ relevant to this example?

 It might be worth reflecting on the new emphasis on patient reported experiences in NHS strategic plans.   

*Waiting in adolescent mental health care*

 “… to the pressures that adolescents face.”

 I didn’t know what Sturm and Drang meant – it’s not common parlance.

 I’m not convinced that this vignette is about care practices that require waiting – the main thing the reader notices from the story is that Layla was seen too late (a counter argument to care practices that need waiting). Is it about Layla needing continuing opportunities to form a therapeutic connection (i.e. may appear inefficient but is actually essential to care)? (e.g. https://bmcprimcare.biomedcentral.com/articles/10.1186/s12875-024-02569-0) If so, I think that needs to be made more explicit.

*Caring on: timely and untimely care*

 I think this section needs a little more balance to acknowledge what is mentioned in the beginning, that some conditions/symptoms requires very timely care, while others requires relational, flexible care. It’s not about putting aside intentions to reduce waiting lists – these performance measures are still extremely important – but it is about how to expand what we consider to be good care and that sometimes good care can happen while waiting.

*Additional comment*

 There is also work around waiting and seeing related to GPs and possible child maltreatment and all the care actions that might entail https://bmjopen.bmj.com/content/3/12/e003894

Is the work clearly and accurately presented and does it cite the current literature?

Yes

If applicable, is the statistical analysis and its interpretation appropriate?

Not applicable

Are all the source data underlying the results available to ensure full reproducibility?

No source data required

Is the study design appropriate and is the work technically sound?

Yes

Are the conclusions drawn adequately supported by the results?

Partly

Are sufficient details of methods and analysis provided to allow replication by others?

Yes

Reviewer Expertise:

Child and adolescent health and wellbeing

I confirm that I have read this submission and believe that I have an appropriate level of expertise to confirm that it is of an acceptable scientific standard, however I have significant reservations, as outlined above.

# Reviewer report: Reviewer response for version 1

**DOI:** 10.21956/wellcomeopenres.24851.r98986

**Published:** 2024-09-27

**Authors:** Ingrid Holme

**Affiliations:** 1 University of Galway, Galway, County Galway, Ireland

Thank you for inviting me to review this fascinating paper, and I have no hesitation in supporting its indexing.

 Waiting is an increasingly problematic issue in many healthcare systems due to a growing demand for services and increasing expectations of immediate action and change. This paper provides deep insight into these factors in a British context and across three specific situations: general practice, mental health, and trans and gender-questioning young people.  There has been extensive investment in research ‘avoidable waiting’, such as in Emergency departments, and a growth in Lean methodology, where waiting is considered a waste.  The three cases covered in this paper clearly show how waiting has other, potentially multiple values.  In emergency departments, dedicated staff are hired to govern workflow and wait times, which are supported by technology (i.e., Symphony); waiting in other medical situations is less clearly managed.  Rather than ‘bedside medicine, ’ most patients experience waiting while samples of their body are sent through biomedical workflows.  In emergency departments, lacking windows and other visible signs of time’s passing, waiting for attention is a collective experience, at least in Irish hospitals ( https://www.irishnews.com/news/northern-ireland/emergency-department-staff-at-breaking-point-as-figures-show-worst-waiting-times-on-record-for-december-SV3Y3AH4WBAMTNUBVJ2L5SYWPE/). 

 The paper is well-written and includes sufficient detail to follow the ethnographic evidence presented. However, readers less familiar with qualitative methodologies might struggle to understand the nuances presented.

 The concept of fugitive care is useful and within the limit of this article, relevant content is provided.  One additional concept which might have use is that of active waiting (i.e. https://www.bodywhys.ie/active-waiting-2/).  Rather than denoting a spiritual commitment to being ‘in the moment’, the healthcare use refers to tasks and activities the patient can undertake during the referral period, a type of patient multitasking.  In regard to the three vignettes presented in the paper, the elongated time involves both the patient and healthcare professional in a socially constructed interaction where pausing is intentional and purposeful (i.e. case 3).  Further exploration of power dynamics and interplays would be fruitful, including exploring how patients discharge themselves by not attending follow-up appointments (as potentially a ‘waste of their time’).  In the end, the paper returns to questions about the relevance of waiting to the NHS and, in my view, suggests a further need to question what waiting looks and feels like with the expansion of virtual wards and digital medicine.  In this regard, the paper raises interesting questions for future research.

Is the work clearly and accurately presented and does it cite the current literature?

Yes

If applicable, is the statistical analysis and its interpretation appropriate?

Not applicable

Are all the source data underlying the results available to ensure full reproducibility?

No source data required

Is the study design appropriate and is the work technically sound?

Yes

Are the conclusions drawn adequately supported by the results?

Yes

Are sufficient details of methods and analysis provided to allow replication by others?

Partly

Reviewer Expertise:

Medical Sociology

I confirm that I have read this submission and believe that I have an appropriate level of expertise to confirm that it is of an acceptable scientific standard.

# Reviewer report: Reviewer response for version 1

**DOI:** 10.21956/wellcomeopenres.24851.r96453

**Published:** 2024-09-17

**Authors:** Olivia J Fisher

**Affiliations:** 1 Wesley Research Institute, Auchenflower, Queensland, USA

Thank you for inviting me to review this high quality and interesting case study. The reconceptualization of “waiting” as a necessary and important component of good healthcare practice is an important addition to the health management literature. The distinction between wasteful waiting and purposeful timely waiting is interesting and has potential applications for service delivery.

 Overall, I found the paper to be well-written and I can see the value of the reflective style of this piece as the authors acknowledge their own experience and learnings. However, I am unclear about how fugitive care is being used as a guiding concept. The definition and description of fugitive care need more clarity for a reader familiar with health service delivery but unfamiliar with the concept of fugitive care. I have a few other minor comments below:

- The abstract is missing the “so what” – is there a key message or learning that this paper is trying to get across? What is the learning that you would like others to know? It seems implied that ‘untimeliness’ can be timely, requiring a reframing of the perception and measurement of timely care but this is not clearly spelled out.
- The concept of fugitive care needs greater explanation and more fleshing out. I am not clear from the text what fugitive care is and how that might differ from “workarounds” in implementation science or distributed or substituted care beyond what is normally provided in a traditional clinician relationship. The case studies do not explicitly describe what is fugitive about the care being provided as compared with usual care, so it is not clear to me as I read the paper why these are being demonstrated as examples of fugitive care. I think a stronger and more explicit link can be made that acknowledges the intersectionality and inequities of waiting and the ways that waiting interacts with fugitive care.   
- The point about witnessing as an act of waiting is well made.
- The conclusory paragraph centres this work within real-world tensions between time as a finite resource and the drive for efficiency, through which waiting is perceived as waste, versus the power of working with timely waiting. This is a strong point which I believe could be more effectively incorporated throughout the paper.
- It would be interesting to consider what are the elements of timely versus wasteful waiting and how health services could be guided to differentiate between the two in the planning and provision of care – just a thought for future research.

Is the work clearly and accurately presented and does it cite the current literature?

Partly

If applicable, is the statistical analysis and its interpretation appropriate?

Not applicable

Are all the source data underlying the results available to ensure full reproducibility?

No source data required

Is the study design appropriate and is the work technically sound?

Yes

Are the conclusions drawn adequately supported by the results?

Yes

Are sufficient details of methods and analysis provided to allow replication by others?

Yes

Reviewer Expertise:

Health services research, implementation science, mental health

I confirm that I have read this submission and believe that I have an appropriate level of expertise to confirm that it is of an acceptable scientific standard, however I have significant reservations, as outlined above.
